# Incidence and factors associated with outcomes of uterine rupture among women delivered at Felegehiwot referral hospital, Bahir Dar, Ethiopia: cross sectional study

**DOI:** 10.1186/s12884-018-2083-8

**Published:** 2018-11-16

**Authors:** Dawud Muhammed Ahmed, Tesfaye Setegn Mengistu, Aemiro Getu Endalamaw

**Affiliations:** 1 Obstetrics and Gynecology, Bahir Dar University, College of Medicine and Health Sciences, P. O box: 79, Bahir Dar, Ethiopia; 2 Bahir Dar University College of Medicine and Health Sciences, Bahir Dar, Amhara Regional State Ethiopia; 3 Marie Stops International Ethiopia, Bahir Dar, Ethiopia

**Keywords:** Ruptured uterus, Felegehiwot referral hospital, Undesired outcomes

## Abstract

**Background:**

Maternal mortality is a major public health challenge in Ethiopia. Uterine rupture is an obstetrical emergency with serious undesired complications for laboring mothers resulting in fatal maternal and neonatal outcomes. Uterine rupture has been contributing to high maternal morbidity and mortality. However, there is limited research on the factors and management outcomes of women with uterine rupture. Understanding the factors and management outcomes might delineate strategies to support survivors. Therefore the aim of this study is to assess the incidence and factors associated with outcomes of uterine rupture among laboring mothers at Felegehiwot Referral Hospital in Bahir Dar City, Northwest Ethiopia.

**Methods:**

This is a cross sectional study with retrospective facility based data collection technique. All pregnant women who were managed for ruptured uterus at Felegehiwot referral hospital from September 11 2012 to August 30 2017 were included. The chart numbers of the women collected from operation theatre registers. Their case folders retrieved from the medical records room for analysis. Using structured check list, information on their sociodemography, booking status, clinical features at presentation and the place of attempted vaginal delivery was extracted. Data on the intraoperative findings, treatment, and associated complications and outcomes also collected. The collected data cleaned, coded and entered into EPI- Info version (7.1.2.0) and then exported in to SPSS Version 20.0 for analysis. Statistical comparison was done using chi square (X^2^). Strength of association between the explanatory variables and outcome variables described using odds ratio at 95% CI and *P* value less than 0.05. The results presented in tables.

**Results:**

We studied 239 cases of uterine rupture in the 5 years period. Mothers without previous cesarean delivery including eight primigravidas took 87% of the cases. From all study participants, 54 of mothers (22.6%) developed undesired outcomes whereas 185(77.4%) discharged without major sequel. More than half (56.9%) arrived in hypovolemic shock. Total abdominal hysterectomy was the commonest procedure accounting for 61.5%. Duration of surgery was less than 2 h in 67.8% of the procedures. Anemia is the commonest complication (80.3%) followed by wound infection and VVF (11.7% each). There were 5 maternal deaths (2.1%). Mothers who had prolonged operation time (> 2 h) (AOR: 2.2, 95% CI: 1.10, 4.63) were significantly associated with undesired maternal outcomes after management of uterine rupture.

**Conclusion:**

Incidence of ruptured uterus and its complications were high in the study area. It reflects the need for improvement in obstetric care and strong collaboration with referring health facilities to ensure prompt referral and management.

## Background

Uterine rupture is tearing of the uterine wall either partially or completely during pregnancy or delivery. This leads to extrusion of the fetus and /or placenta in to the maternal abdomen and massive hemorrhage especially when the rupture is of unscarred uterus [1–4], Uterine rupture contributes significantly to both fetal and maternal mortality, serous morbidities and loss of fertility from hysterectomy. The severity of fetal and maternal morbidity depends on the extent of uterine rupture [1, 5–7]. There is wide variation in incidence between developed and developing countries. In developing countries [8] the incidence is high due to socio economic factors, cultural practices and lack of access to antenatal and intra-partum care. This can be evidenced by the greater number of un-booked obstetric emergencies, often originating from rural areas with poor antenatal care [9], poor obstetric care [10, 11], few comprehensive emergency care facilities [2, 12], and poor socioeconomic status of the community [6, 13].

In Ethiopia for every 1000 births there are about 4 maternal deaths [14]. Uterine rupture with or without obstructed labor is the leading cause of maternal mortality accounting for 36% of the total maternal mortality [15]. This may be a reflection of high prevalence of home delivery [5, 15], especially in the rural areas. Majority of pregnant women in Ethiopia stay at home laboring for 2–3 days and come to health facility when they are seriously ill [15].

Previous caesarean section had been one of the leading cause of uterine rupture in developed countries, while uterine rupture from unscarred uterus is more prevalent in less and least developed countries [1, 7]. Studies conducted in the developing world give strong evidence that uterine rupture is a major health problem in these countries with the rate being high in rural areas [13]. A major factor in uterine rupture in developing countries is obstructed labour due to inadequate access to medical care [2, 3, 16, 17]. Also the high incidence of contracted pelvis among black African women is found to be a high risk factor for obstetric complications. Other risk factors for uterine rupture include grand multiparity, instrumental delivery, and use of uterotonic drugs to induce or augment labour [5, 13]. Rarely placenta percreta and intrauterine manipulations such as internal podalic version and breech extraction can result in uterine rupture [1].

The type of surgical intervention on the uterus is dependent on the type and extent of rupture, hemodynamic status of the mother, desire for future fertility, presence of gross infection and experience of the surgeon. This could be total or subtotal abdominal hysterectomy, uterine repair with or without tubal ligation [3, 17–20]. Uterine repair should be reserved for women who have low transverse rupture, no extension of the tear to broad ligaments, cervix or vagina, easily controllable hemorrhage, good general condition, desire for future child bearing and no evidence of gross infection. Hysterectomy is appropriate for those with one of the above intra operative findings [17].

Uterine rupture is associated with a number of acute and long term complications. These include anemia, need for transfusion, bladder injury, wound infection, sepsis and death [19]. Complications like obstetric fistula, foot drop, psychological trauma, permanent loss of fertility are some of the long term outcomes [17, 18].Acute renal failure from pre renal azotemia is also possible following massive hemorrhage [9, 21].Among these, the most commonly encountered complication is hemorrhage leading to anemia [3, 5, 18, 19].

Not only this, Loss of fertility in communities where reproduction is considered the very essence of womanhood has grave socio cultural implications like divorce, and loss of economic support [3, 18].

Patients with fistula are living leaking urine or feces through the vagina. They have to continue living thereafter unclean, outcast, smelling of urine and faces. This is a cause for separate from their families, worsening poverty, malnutrition and almost unendurable suffering [22].

Maternal death as a consequence of uterine rupture occurs at a rate of 0–1% in modern developed nations, but the mortality rates in developing countries are 5–10% [17].

The determinant factors for maternal outcome of uterine rupture differ across geographical boundaries due to the difference in socio-demographic status, and the availability and accessibility of skilled birth attendant and health system effectiveness [5].

Once diagnosis of uterine rupture is entertained, the time spent for successful surgical intervention should be very short and all available resources should be quickly mobilized for favorable outcome of both mother and new born [17, 18].Late arrival and treatment increase the maternal and fetal morbidity and mortality [18]. Difficulty performing an exact diagnosis at the arrival of the patient with a severe diagnosis like uterine rupture may worsen the condition [13]. Immediate surgery and blood replacement plays major role in maternal survival [13, 18]. The main causes of maternal mortality in rupture uterus are failure to diagnose the condition at the first referral center and arrival at the tertiary center in a moribund condition [23]. The immediate cause of death in such condition includes puerperal sepsis [18] and hypovolemic shock [19].

Prognosis for the fetus is even worse than the mother [18]. Studies in different parts of developing countries indicated high fetal case fatality rate [2, 5, 18, 24];complete uterine rupture being associated with the highest fetal death rate [18].

Initiation of labor at health institutions, early referral [2], and treatment of hypovolumia and prevention of postoperative anemia is recommended to decrease maternal death secondary to uterine rupture [5, 24]. In addition proper monitoring of labour and improvement of comprehensive emergence obstetric care at all levels of health care are recommended to avoid unnecessary delays in care [3].Early diagnosis and active surgical management will go a long way in reducing maternal and fetal mortality [9].

Uterine rupture remains an important clinical problem in northern Ethiopia. Changes in the cultural preference for home delivery, better transport and referral systems, and improved obstetric training and hospital management of laboring women are needed [20].

Currently the federal ministry of health of Ethiopia is improving obstetric care by scaling up of skilled personnel attended delivery; universal primary education, infrastructure and human resource development [15, 20, 25]. Despite these strategies births assisted by skilled provider in our country is 28% [14]. This indicates the need for further research and implementation of programs to improve health status of the country.

### Statement of the problem

The incidence of uterine rupture has regional and sub-regional variability posing a major public health problem in under developed countries [1, 7]. The rate of pregnancy-related uterine rupture in women with unscarred uterus is 0.012% (1 in 8434) for women living in industrialized countries and 0.11% (1 in 920) for women living in developing countries [17].

In low resource settings multiple factors including educational status of women, ignorance, poverty, regular antenatal care checkup, home delivery, prolonged (dysfunctional) and obstructed labor had been identified risk factors of uterine rupture [21, 26]. Similarly evidences showed that previous cesarean delivery, mal-presentations [7, 27], induction and augmentation of labor [27], grand multiparty [7, 28], neglected labor, breech extraction, uterine instrumentation are predisposing factors for uterine rupture [7].

Evidences showed that abdominal pain, vaginal bleeding, loss of fetal station, non-reassuring fetal heart rate, shock and fetal bradycardia are most common indicators of uterine rupture [29]which needs prompt diagnosis and treatment before progressing serious maternal and perinatal outcomes [7, 30, 31]. Hemorrhage [7, 31], hypovolemic shock, need for blood transfusion [31], bladder injury, need for hysterectomy, and a maternal death are some of maternal consequences/outcomes while, admission to neonatal intensive care unit, fetal hypoxia or anoxia, and neonatal death are some of neonatal outcomes [7]. However, maternal/neonatal morbidity and mortality following uterine rupture depend on the level of medical care [7].

Although uterine rupture is preventable condition, it has also been one of the leading cause of maternal mortality in Ethiopia [15, 26]. The federal ministry of health is providing curative health services to the community as one of the priorities, and, as a consequence, the number of government hospitals in the country has increased from 126 to 31. It is also trying to change the cultural preference of home delivery through better transport, access to prenatal care, obstetric training and exempted service charges for laboring mothers (2014 FMOH Bulletin). As part of this improvement Bahir dar university launched residency programs in Obstetric in 2014 using Felege hiwot referral hospital as affiliated site. There is a 24 h blood transfusion service and intensive care unit in the hospital. Despite all these, evidences are lacking as to the magnitude and factors for the outcome of uterine rupture in this area. In addition there was no effort to measure changes in management outcome as a result of improvement of obstetric services. Therefore the aim of this study is to identify factors associated with outcomes of uterine rupture among laboring mothers at Felegehiwot Referral Hospital in Bahir Dar City, Northwest Ethiopia.

### Literature review

Uterine rupture has continued to be a catastrophic feature of obstetric practice especially in the low-resource settings [28].

There are some studies conducted in Ethiopia. Among these a prospective study over a period of 2 years at Debre Markos hospital found the incidence of ruptured uterus to be 3.8%. Susceptible groups were age 25 to 29 years and grand multiparas (> 5). Complete type uterine rupture accounted for 88.6%. In more than half (54.3%) of the cases the site of rupture was anterior. The commonest procedure performed was hysterectomy (81.4%). The postoperative complication rate was 24.3%. Sepsis was the leading cause of death [16].

But more recently a review of uterine rupture cases at Ayder Referral hospital in Mekelle, northern Ethiopia between 2009 and 2013 found a rate of uterine rupture of 1 in 110 deliveries. Predisposing factors in order of their frequency were cephalopelvic disproportion (74%), previous cesarean delivery (11%), and fetal malpresentation (9%). The presenting symptoms were pain, sudden cessation of contractions during labor and vaginal bleeding. The vast majority of ruptured uterus occurred from unscarred uterus; only five patients had undergone a previous cesarean delivery. The mean gravidity was 3.6. Less than two-thirds had received any prenatal care. Almost all patients came to Ayder from referring health facilities. One patient who had unrecognized ureteric injury and persistent anemia died. Perinatal mortality was 94% [20].

Analysis of the causes, complications and management outcomes of ruptured uterus in Dar-es- Salaam, Tanzania shows the incidence to be 2.25 per 1000 deliveries. The leading causes identified were obstructed labor, previous cesarean delivery and use of uterotonic drugs for induction and augmentation of labor. Most of these patients were referrals from municipal hospitals and all attended antenatal follow up at least once. There were 21 maternal deaths and 157 perinatal deaths giving case fatality rate of 12.9 and 96.3% respectively. The commonest maternal complication was hemorrhage (34.4%) followed by sepsis, VVF and blood transfusions. Subtotal hysterectomy is the most common performed procedure (73.6%), repair with BTL (12.3%), repair only (12.3%) and total hysterectomy (1.8%). Most operations were made by obstetricians [3].

Rupture of the gravid uterus is still a significant cause of maternal mortality and morbidity in Nnamdi Azikiwe University Teaching Hospital, Nnewi, Anambra State Southeast Nigeria. The incidence was 1 in 161 deliveries. The commonest age range of occurrence was 30–34 years. Contrary to widespread belief that uterine rupture is a disease of multiparous women, in this study women of low parity predominate. Most of the ruptures were as a result a combination of risk factors like previous caesarean section with concurrent use of oxytocic. The commonest procedure performed was uterine repair only [32].

A 10 year retrospective study in the same hospital showed an incidence of 0.84%. All the patients were multiparous and 63.8% were unbooked. Majority were Traumatic (iatrogenic) ruptures (72.1%). Uterine repair with (55.8%) or without (34.9%) bilateral tubal ligation was the commonest surgery performed. The case fatality rate and perinatal mortality rate were 16.3 and 88.4% respectively. Average duration of hospitalization following surgery was 10.3 days [28].

There is also a retrospective study describing the factors influencing the management and the prognosis of ruptured uterus in a level III maternity care center of a third world country (Cocody University Hospital Center, Abidjan-Cote d’Ivoire). There were 513 cases of ruptured uterus between January 2002 and December 2014 giving an incidence of 0.95%. Most cases occurred in women with unscarred uterus (76.8%). Radical hysterectomy was done in 35.3% of all women. Uterine repair was done more commonly for women from the communes of Abidjan and its suburbs (71%). Maternal mortality rate was 5.8% and factors like the type of surgery (*p* = 0.000), the time of uterine rupture (*p* = 0.000) and the transportation distance (*p* = 0.000) were significantly associated. Fetal mortality was 94.1% [8].

Study done in Sweden showed increased risk of uterine rupture (during their second delivery) in women who underwent a cesarean delivery compared with women who delivered vaginally in their first birth. Additional factors associated with increased risk of uterine rupture were induction of labor, high (> or = 4000 g) birth weight, post term (> or = 42 weeks) births, high (> or = 35 years) maternal age, and short (< or = 164 cm) maternal stature [33].

Researchers found the incidence of ruptured uterus to be 0.116% in one of the tertiary care hospital in Turkey. Trial of labor after cesarean was the most common cause of uterine rupture accounting for 31.1% of the cases. Vaginal Bleeding was the main symptom at presentation (44.3%). Lower uterine segment (isthmus) was the most vulnerable part of uterus (39.3%) for rupture. Women with delayed surgical intervention and older patients with increased parity were likely to have longer hospitalization periods [34].

One research in Zurich with special interest on effect of uterine fundal pressure on uterine rupture revealed previous uterine surgery as the main risk factor for uterine rupture in the whole study population. Risk factors in women with unscarred uterus were uterine fundal pressure (UFP), abnormal placentation, and age at delivery > 40 years. The only factor which can be modified is Uterine Fundal Pressure [35].

Population based study in Sweden reported the overall rate of uterine rupture among women with an attempted vaginal birth in their second delivery to be 0.91/1000. The rate of uterine rupture among women who attempted vaginal birth after a caesarean section was 9.00/1000 compared with a uterine rupture rate of 0.18/1000 among women without a history of caesarean delivery. Compared with women who experienced a spontaneous onset of delivery, women whose second delivery was induced faced a doubled increase in risk of uterine rupture. Induction of labor was associated with a doubled risk of uterine rupture both among women with a previous caesarean and among women who did not have previous caesarean. High maternal age, induction of labour, and high birth weight increased the risk of uterine rupture. The neonatal death rate was 51.09/1000. This was more than 60 fold increase compared with neonatal death rate among women without uterine rupture (1.4/1000) [36].

A population based study aimed at determining trends, risk factors and pregnancy outcome in women with uterine rupture compared all singleton deliveries with and without uterine rupture between 1988 and 2009. Uterine rupture occurred in 0.06% of all deliveries; 59% in women with a previous cesarean delivery. There was a gradual increase in the rate of uterine rupture from 1988 (0.01%) to 2009 (0.05%). Independent risk factors for uterine rupture were: previous CD, preterm delivery (< 37 weeks), malpresentation, parity, and dystocia during the first and second stages of labor. In addition, Uterine rupture was noted as an independent risk factor for perinatal mortality [37].

Rupture of gravid uterus brings about potentially hazardous risks. Regular antenatal care, hospital deliveries and vigilance during labor with quick referral to a well-equipped center may reduce the incidence of this condition [34]. There is therefore a dire need for education of women on health-related issues, utilization of available health facilities, adequate supervision of labour and provision of facilities for emergency obstetric care [28].

Uterine rupture is a complication that can be eliminated under conditions of best obstetric practice. To attain this objective, use of misoprostol in primary health facilities should be stopped or proper management of the medication instituted. The survival of patients after uterine rupture depends on the time interval between rupture and intervention, and the availability of blood products for transfusion [38].

### Justification of the study

Maternal and fetal morbidity and mortality is high in Ethiopia. Ruptured uterus contributes significantly to maternal mortality. In spite of this, there has been paucity of evidences on factors associated with management outcome of mothers who had uterine rupture. This study documents common complications and factors related with management outcomes of uterine rupture in FHRH. Therefore, the result of this study helps policy makers, program, planners, governmental and non-governmental organization implementers and maternal health service providers/practitioners to provide evidence based interventions which will contribute in maternal morbidity and mortality reduction in the hospital and the region as well. Most importantly, since there are limited research evidences on uterine rupture in the study area, this study serves as a baseline work for other researchers interested to work on the risk factors and outcomes of women who had uterine rupture.

### General objectives

The objective of the study is to assess the incidence of uterine rupture and factors associated with outcomes of uterine rupture among women delivered in Felegehiwot referral hospital in Bahir Dar City, Northwest Ethiopia.

### Specific objectives

- To determine the incidence of uterine rupture among women delivered at Felege hiwot referral hospital in the study period
- To identify complications after management of uterine rupture
- To identify factors associated with outcome of clients managed for uterine rupture in FHRH

## Methods

### Study area and period

Institutional based cross-sectional study was conducted from May 1 to 30, 2017 in Bahir Dar City, Felege Hiwot Referral Hospital. Bahir Dar is the capital city of Amhara National Regional State (ANRS), located 565 km Northwest of Addis Ababa. FHRH is one of the 42 governmental Hospitals in Amhara Regional state. The hospital serves for more than 5,000,000 populations in its catchment area (ANRHB 2015). The hospital has one big maternity ward which possesses around 74 beds. There are about 6000 deliveries per year, 30% of which is cesarean deliveries (prenatal report, 2016). There are 7 obstetricians, 27 residents and 25 midwives currently working in the department of obstetrics. As it is a referral hospital most of the clients are referral cases from health centers and district hospitals.

### Study design

Cross sectional study with retrospective facility based data collection was used.

### Source population

Women who had uterine rupture (reached through their charts documented during the procedure).

### Study population

Women who had uterine rupture in the time from September 11 2012 to August 30 2017 were included.

### Inclusion and exclusion criteria

#### Inclusion criteria

All women who had diagnosed with uterine rupture and managed at FHRH from 2012 to 2017 were included.

#### Exclusion criteria

Women who had uterine rupture and managed at other health facilities and referred to FHRH for complications like transfusion, ICU admission and Women who had ruptured uterus from medical termination of pregnancy in the second trimester, and those cases with missed data on outcome variable and major factors (parity, age, duration of labor, place of labor, operation factors…) were not included in the study.

### Sample size determination

A single population proportion formula using the assumptions of 95% confidence level and 5% margin of error was used to estimate the sample size. Estimated proportion of laboring at home among ruptured cases i.e. 57% (*P* = 0.57) was used (Astatikie et al. [5]). Substituting the above assumption in the formula, the required sample size is calculated to be 376.

$$ \mathrm{n}=\underline {{\left(\mathrm{Z}\upalpha {/}_2\right)}^2\mathrm{xp}\left(1\hbox{-} \mathrm{p}\right)/}{\mathrm{d}}^2 $$; Where

n = Sample size

Z a/_2_ = Confidence level at 95% = **1.96**

P = Proportion of population which is 57%

**Table:** 

Factor	Proportion	Sample size	Reference
Labor at home	57%	**376**	Astatikie G et al. 2017 [5]
Hypovolemic shock	34.3%	346	“
Post op anemia	21.9%	262	“

### Sampling procedure

First the main registration book of the Operation room where all emergencies registered according to their order was used to list all uterine rupture cases. Each of the cases included. The names of the mother’s selected were checked from the charts and data was collected daily.

### Study variables

**Dependent Variable:**

Outcome of uterine rupture

**Independent variables**

- **Socio-demographic variables:** age, address
- **Obstetric factors:** Parity, ANC status, previous cesarean delivery, obstructed labor, referral from health facility, induction and augmentation, place of trial of labor, blood pressure at arrival, anemia, duration of labor, type of rupture, type of surgical procedure and duration of surgery.

### Measurement

The outcome variable was measured as desired outcome and undesired outcomes. The desired outcome was also measured as cured without major sequel (Yes, No). Women were classified as having undesired management outcome when she had developed one of the following condition: death, permanent organ injury, obstetric fistula, wound dehiscence, Sepsis, ICU admission.

Maternal death secondary to uterine rupture is defined as death of the mother from uterine rupture, its complications or management.

Sepsis: a woman will be declared she has sepsis if she is diagnosed and treated as recorded in the patient chart.

Operation time is defined prolonged if it took more than 2 h.

Senior resident is a physician who is on fourth year of training in gynecology and obstetrics.

Source of admission is recorded as it is written from admission note.

Time of arrival from 7:00 AM to 6:59 PM is labeled as “Day time”, and 7:00 PM to 6:59 AM as “Night time”.

### Data collection

The folder numbers of the women who were managed for ruptured uterus over a 5 year period (September 11 2012–August 30 2017) was collected from operation theatre registers. Their case folders were retrieved from the medical records department. Using structured check list, information on their sociodemography, booking status, clinical features at presentation and the place of attempted vaginal delivery was extracted by the data collectors. Data on the intraoperative findings, treatment, associated complications and maternal outcomes were also collected. Check list checked by data collectors & supervisors on daily base for completeness.

### Data processing and analysis

Data was entered in to Epi Info version 7.1.2.0 and then transported to SPSS version 20 software packages for analysis. Descriptive statistics such as mean, percentage and standard deviation, was determined. Bi variable logistic regression was used to determine the association between each independent variable and the outcome variable. The degree of association between dependent and independent variables was determined using the OR with CI of 95% and *p*-value of < 0.05.

### Data quality control

Prior to data collection, the check list was tested to check the consistency of the checklist format and the ability of the data collector’s performance. The checklist was modified based on the pretest results. One day training/ orientation how to carry out their duty was given for the data collectors.

## Results

There were a total of 262 cases of uterine rupture at FHRH in 5 years period from 2012 to 2017. The total number of deliveries in the same period was 28,835. This gives an incidence of 0.9% (1 in 110). Among the uterine rupture cases, 10 charts were lost from card record room and additional 13 were rejected because of missing information. The final study population becomes 239. Response rate becomes 91%.

### Socio demographic characteristics of study participants

Most of the study population (95.8%) was in the age group of 17–39. The mean age is 29.38 years. Most came from areas outside Bahir dar, Debub Gondar being the highest (36.4). There were 2 cases from Benshangul Gumuz region (Table 1).

**Table 1:** Socio demographic characteristics of uterine rupture in Felegehiwot Referral hospital from September 2012 to August 2017(*n* = 239)

Variables	Frequency	Percent
Age of study participants		
< =16	0	0.0
17–39	229	95.8
> 40	10	4.2
Total	239	100.0
Address of study participants		
Awi	69	28.9
Bahir dar Zuria	28	11.7
Metekel zone	2	0.8
West Gojjam	53	22.2
South Gondar	87	36.4
Total	239	100.0

### Obstetric variables

Most are multigravidas with only eight uterine rupture cases among Primigravidas (3.3%). Most of the ruptures occurred on unscarred uterus (87%). Half of respondents have four and above Antenatal visits. 78% have attempted delivery at health centers. Ten uterine ruptures occurred at Felege Hiwot hospital. Uterine rupture was diagnosed correctly in 17.6% of cases up on referral. Most arrived with hypovolemic shock (Table 2).

**Table 2:** Obstetric variables of uterine rupture in Felegehiwot Referral hospital from September 2012 to August 2017(*n* = 239)

Variables	Frequency	Percent
Gravidity		
Primi gravida	8	3.3
Multi gravida	157	65.7
Grand multi gravida	74	31.0
Total	239	100.0
Gestational age		
Preterm	16	6.7
Term	164	68.6
Post term	30	12.6
Unknown	29	12.1
Total	239	100.0
Antenatal care		
No	53	22.2
< 4	68	28.5
> 4	118	49.4
Total	239	100
Previous cesarean delivery		
Yes	31	13
No	208	87
Total	239	100
Source of admission to FHRH		
Home	14	5.9
health center	188	78.7
district hospital	27	11.3
FHRH	10	4.2
Total	239	100.0
Duration of labor		
< 24 h	191	79.9
24–48	41	17.2
> 48	7	2.9
Total	239	100.0
Diagnosis on referral		
uterine rupture	42	17.6
CPD/OL	94	37.6
Second stage	31	13.0
IUFD	28	11.7
other	23	9.6
No referral	21	8.8
Total	239	100.0
Time of arrival		
Day	164	68.6
Night	75	31.4
Total	239	100.0
Blood pressure on arrival		
Non recordable	53	22.2
< 90/60mmhg	83	34.7
> 90/60mmhg	103	43.1
Total	239	100.0
Diagnosis on admission to FHRH		
uterine rupture	202	84.5
other diagnosis	37	15.5
Total	239	100

### Presenting symptoms

The three symptoms identified were abdominal pain (65.7), cessation of labor (56.5) and vaginal bleeding (45.65). The commonest presenting sign of uterine rupture is uterine tenderness (81.2%). Fetal heart sounds were positive in 12.6% of cases (Table 3).

**Table 3:** Clinical presentations among cases of uterine rupture in Felegehiwot Referral hospital from September 2012 to August 2017(*n* = 239)

Symptoms	Frequency	Percent
1. Abdominal pain	157	65.7
2. cessation of labor	135	56.5
3. vaginal bleeding	109	45.6
4. tenderness	194	81.2
5. easily palpable fetal parts	132	55.2
6. uterine contraction present	34	14.2
7. FHB positive	30	12.6
8. Palpable defect on uterus	56	23.4

### Intra operative findings

Anterior lower segment is the commonest site of uterine rupture accounting for 56%. Total abdominal hysterectomy (61.5%) leads from procedures performed. There were 8 bladder ruptures diagnosed intraoperative (Table 4).

**Table 4:** Operative findings among cases of uterine rupture in Felegehiwot Referral hospital from September 2012 to August 2017(*n* = 239)

Intra operative findings	Frequency	Percent
Type of rupture		
Complete	203	84.9
Incomplete	36	15.1
Total	239	100.0
Site of rupture		
Anterior lower segment	134	56.1
Posterior	19	7.9
Lateral	78	32.6
Fundal	8	3.3
Total	239	100.0
Bladder injury	8	3.3
Necrotic edges	55	23.0
Vaginal extension	64	26.8
Duration of surgery		
< =2 h	162	67.8
> 2 h	77	32.2
Total	239	100.0
Type of Procedure		
TAH	147	61.5
STAH	24	10.0
repair only	54	22.6
repair with BTL	14	5.9
Total	239	100.0
Surgeon		
consultant	174	72.8
resident	65	27.2
Total	239	100.0

### Complications

Anemia is the commonest complication (80.3%) followed by wound infection and VVF. There were 5 maternal deaths (2.1%). Totally there were 54(22.6%) mothers who developed undesired outcomes (Table 5).

**Table 5:** Complications among cases of uterine rupture in Felegehiwot Referral hospital from September 2012 to August 2017(*n* = 239)

Complications	Frequency	Percent
Post op hemoglobin		
> 12 g/dl	47	19.7
7-12 g/dl	169	70.7
< 7 g/dl	23	9.6
Total	239	100.0
Transfusion		
No	59	24.6
1 units	38	16.0
2 units	97	40.6
3 units	24	10.0
4 units	15	6.3
> =5 units	6	2.5
Total	239	100
Wound infection	28	11.7
Hospital stay		
< 8 days	160	66.9
> =8 days	79	33.1
Total	239	100.0
Undesired outcomes		
ICU admission	5	2.1
VVF	28	11.7
Sepsis	12	5.0
Wound dehiscence	15	6.3
Death	5	2.1

Perinatal outcome: 84.1% of mothers end up in still birth. In 16 charts nothing is documented about the status of the neonate.

### Factors associated with outcomes of uterine rupture

In multi variable logistic regression analysis it is found that mothers with operation times more than 2 h (AOR: 2.260, 95% CI: 1.102, 4.638) were more likely to have undesired maternal outcomes than operation times less than 2 h. Nearly one third (31.2%) of those cases whose operation time was more than 2 h, resulted in undesired outcomes. Gravidity, gestational age, place of attempted delivery, and time of arrival to Felegehiwot were not significantly associated with maternal outcomes (Table 6).

**Table 6:** Binary and multivariable logistic regression table for factors associated with outcome of uterine rupture in Felegehiwot hospital from September 2011 to August 2017(*n* = 239)

Variables	UR management outcomes		COR (95% CI)	AOR (95% CI)
	Undesired n (%)	Desired n (%)		
Gravidity				
Primigravida	4 (7.4%)	4 (2.2%)	1.0	1.0
Multigravida	26 (48.1%)	131 (70.8%)	0.2 (0.04–0.84)	0.2 (0.03, 1.30)
Grandmultigravida	23 (42.6%)	45 (24.3%)	0.5 (0.11–2.23)	0.7 (0.11, 4.64)
Gestational age				
Preterm	5 (9.3%)	11 (5.9%)	1.0	1.0
Term	25 (46.3%)	139 (75.1%)	0.3 (0.12–1.23)	0.3 (0.08, 1.17)
Post term	8 (14.8%)	22 (11.9%)	0.8 (0.21–3.02)	0.5 (0.11, 2.46)
Unknown	16 (29.6%)	13 (7.0%)	2.7 (0.74–9.79)	2.6 (0.63,11.52)
Place of attempted delivery				
Home	6 (11.1%)	8 (4.3%)	1.0	1.0
Health Center	40 (74.1%)	148 (80.0%)	0.3 (0.11, 1.09)	0.6 (0.16, 2.47)
District hospital	8 (14.8%)	19 (10.3%)	0.5 (0.14,2.15)	0.8 (0.17, 4.24)
Time of arrival to FHRH				
Day	43 (79.6%)	121 (65.4%)	1.0	1.0
Night	11 (20.4%)	64 (34.6%)	0.4 (0.23,1.00)	0.4 (0.22,1.08)
Duration of surgery				
< =2 h	30 (55.6)	132 (71.4)	1.0	1.0
> 2 h	24 (44.4)	53 (28.6)	1.9 (1.06, 3.72) ^*^	2.2 (1.10,4.63)

^*^statistically significant at *P* – value < 0.05

## Discussion

The incidence of uterine rupture is 0.9% (1 in 110). This is lower than the 2012 incidence in this same hospital (2.9% or 1 in 35) and the prevalence found at Debre Markos hospital 2.24%(5) [39]. This may be as a result of establishment of functional district hospitals capable of managing these cases. But still it is higher when compared to incidence in other developing countries like 0.22% in Dar-es- Salaam, Tanzania and 0.057% in Imphal, India [3, 40]. This might be a reflection of delay in timely diagnosis of uterine rupture and referral once women reach health centers.

Most of the mothers in this study were referrals from health institutions (90%). This is similar with the study in Dar selam, Tanzania where most patients were referrals from municipal hospitals. Despite the national report of high prevalence of home delivery in Ethiopia [5, 15] especially in the rural areas, in this study it shows only 5.9%. The low figure of home delivery in this study may be a reflection of inappropriate assignment of those with referral papers as if they tried labor at health institution. But the common scenario is visiting health centers when they are seriously ill after laboring for 2–3 days at home. This affects the true figure of the problem. The other possibility may be inadequate supervision of labour and emergency obstetric care. Uterine rupture was diagnosed correctly in only 17.6% of the cases by referring health institution. This may be because of either difficulty in diagnosing uterine rupture or ruptures occur on the way to Felegehiwot.

There were 10 cases of uterine rupture at Felege Hiwot, none of whom developed undesired outcome. This may be due to prompt diagnosis, blood transfusion whenever necessary and immediate laparotomy indirectly reflecting the improved obstetric practice at this referral hospital.

Most underwent Total abdominal hysterectomy (62% TAH), & 10% had STAH. Around 75% of mothers from areas outside Bahir dar underwent hysterectomy whereas half from Bahir dar and its surroundings. This findings are consistent with the finding from Cocody University Hospital Center, Abidjan-Cote d’Ivoire which shows more conservative surgeries by uterine suture in women from the communes of Abidjan and its suburbs (71%) versus 25% of women who came from inland towns [8]. However, in other studies the commonest procedure carried out was uterine repair only [9, 11, 13, 32]. Unstable hemodynamic status, more number of obstructed labors, presence of gross infection, and higher parities in our study may lead to hysterectomy.

Vesico Vaginal fistula (VVF) affected large proportion of mothers (11.7) who sustained uterine rupture similar with other studies [18]. However one study in India reveals a 2% risk [9]. The possible reason for this difference is high rate of obstructed labor in our study area, where as in India majority are following uterine scar.

Blood availability for transfusion as part of management of uterine rupture is lifesaving. In this regard 75.3% of study participants had blood transfusion and 40.6% got at least two units of cross matched blood. This is not usually possible in some countries. Even if all are anemic, only 57.1% of mothers got 500-1000 ml blood transfusion at Al-thawra hospital, the main hospital in Sana’a City, the capital of the Republic of Yemen. This better blood availability in our study may indicate strong commitment of Federal ministry of health and continuous work of student clubs in mobilizing the community to donate blood.

There were 5 maternal deaths (2.1%) as a result of uterine rupture in the study period. This is lower as compared with deaths at Debre Markos hospital (6.6%) and Cot devours (5.8%) [5, 8]. Early diagnosis and management of uterine rupture, widely available blood transfusion services as well as close follow up post operatively may have contributed for this reduction. In this regard the hospital being teaching institution for the residency program, interns and residents could have a significant role.

According to research done at Abidjan, Cote d’Ivoire, maternal mortality was significantly influenced by the type of surgery (*p* = 0.000), time of uterine rupture (*p* = 0.000) transportation distance (*p* = 0.000) [8]. In our study as well, four out of the five maternal deaths were from those treated with hysterectomies. This may be mainly due to the combination of more severe lesions in hysterectomies than repairs.

This study reveals that undesired outcomes were more likely to occur(two times) if time of operation took more than 2 h (AOR: 2.260, 95% CI: 1.102, 4.638). Nearly one third (31.2%) of those cases whose operation time was more than 2 h, resulted in undesired outcome. This may be attributed to the complexity of the lesion in ruptured uterus and poor patient condition.

The age, source of admission, duration of labor before arriving FHRH, site and type of rupture, gestational age, and previous operations were not significantly associated with outcomes of uterine rupture.

The fetal outcome was poor with around 84% of still births. This is similar with other studies in developing countries [11]. There is Poor documentation about the baby that make difficult to analyze other factors.

## Conclusion

This study shows high incidence of uterine rupture with significant undesired outcomes in Northwest Ethiopia. It reflects the need for improvement in obstetric care and strong collaboration with referring health facilities to ensure prompt referral and management.

### Limitations of the study

Since the study is retrospective, 23 charts could not be included in this study because of missed variables and lost charts. Prospective multicenter study in the future will alleviate the limitations of this study.

## Abbreviations

ANC: Antenatal care
ANRHB: Amhara National regional Health Bureau
ANRS: Amhara National Regional State
AOR: Adjusted odds ratio
BTL: Bilateral tubal ligation
CD: Cesarean delivery
CPD: Cephalo pelvic disproportion
FHRH: Felegehiwot referral hospital
FMOH: Federal Ministry of Health
ICU: Intensive care unit
IUFD: Intra uterine fetal death
MD: Medical doctor
MPH: Master of Public health
NICU: Neonatal Intensive Care Unit
OL: Obstructed labor
RH: Reproductive health
SPSS: Statistical package for social sciences
STAH: Sub total abdominal hysterectomy
TAH: Total abdominal hysterectomy
UFP: Uterine fundal pressure
VVF: Vesico vaginal fistula
WHO: World Health Organization
